# The Phenotype of Patients with a Recent Fracture: A Literature Survey of the Fracture Liaison Service

**DOI:** 10.1007/s00223-017-0284-1

**Published:** 2017-05-24

**Authors:** Lisanne Vranken, Caroline E. Wyers, Joop P. W. van den Bergh, Piet P. M. M. Geusens

**Affiliations:** 1 Department of Internal Medicine, VieCuri Medical Centre, P.O. Box 1926, 5900 BX Venlo, The Netherlands; 2 Department of Internal Medicine, NUTRIM School of Nutrition and Translational Research in Metabolism, Maastricht University Medical Centre+ (MUMC+), P.O. Box 616, 6200 MD Maastricht, The Netherlands; 3 Biomedical Research Centre, Hasselt University, Agoralaan, Gebouw D, 3590 Diepenbeek, Belgium; 4 Department of Internal Medicine, Subdivision Rheumatology, CAPHRI, Maastricht University Medical Centre+ (MUMC+), P.O. Box 616, 6200 MD Maastricht, The Netherlands

**Keywords:** Fracture Liaison Service, Fractures, Secondary prevention, Phenotype

## Abstract

The aetiology of fractures in patients aged 50 years and older is multifactorial, and includes bone- and fall-related risks. The Fracture Liaison Service (FLS) is recommended to identify patients with a recent fracture and to evaluate their subsequent fracture risk, in order to take measures to decrease the risk of subsequent fractures in patients with a high risk phenotype. A literature survey was conducted to describe components of the bone- and fall-related phenotype of patients attending the FLS. Components of the patient phenotype at the FLS have been reported in 33 studies. Patient selection varied widely in terms of patient identification, selection, and FLS attendance. Consequently, there was a high variability in FLS patient characteristics, such as mean age (64–80 years), proportion of men (13–30%), and fracture locations (2–51% hip, <1–41% vertebral, and 49–95% non-hip, non-vertebral fractures). The studies also varied in the risk evaluation performed. When reported, there was a highly variability in the percentage of patients with osteoporosis (12–54%), prevalent vertebral fractures (20–57%), newly diagnosed contributors to secondary osteoporosis and metabolic bone disorders (3–70%), and fall-related risk factors (60–84%). In FLS literature, we found a high variability in patient selection and risk evaluation, resulting in a highly variable phenotype. In order to specify the bone- and fall related phenotypes at the FLS, systematic studies on the presence and combinations of these risks are needed.

## Introduction

Fractures constitute a major health care concern worldwide, as 50% of women and 20% of men at the age of 50 years will sustain a fracture during their remaining lifetime [1, 2]. Since the world population is ageing, the annual number of fractures is expected to increase from 3.5 million in 2010 to 4.5 million in 2025, corresponding to an increase of 28% [3].

Fractures indicate an increased risk of subsequent fractures and premature mortality [4–7]. Current guidelines recommend secondary fracture risk evaluation in all men and women aged 50 years and older with a recent clinical fracture [8–11]. However, many fracture patients were not offered appropriate secondary fracture prevention, resulting in a care gap throughout the world [12].

Fracture Liaison Services (FLS) have been designed and implemented to diminish the care gap [13]. The key components and objectives of a FLS are multiple. Firstly, case finding by systematic identification and selection of fracture patients. Second, to adequately evaluate subsequent fracture risk using clinical risk factors for fractures and falls, dual-energy X-ray absorptiometry (DXA) and imaging of the spine for detection of previously unknown vertebral fractures. Third, analysis for eventual underlying secondary osteoporosis and metabolic bone disorders. Fourth, adequate treatment in patients at high risk, and fifth, development of a follow-up program [14].

Unfortunately, FLS are currently established in a small proportion of facilities that receive fracture patients worldwide [15]. The International Osteoporosis Foundation (IOF), American Society for Bone and Mineral Research (ASBMR), European League Against Rheumatism (EULAR), and European Federation of National Associations of Orthopaedics and Traumatology (EFORT) support the implementation of FLS as they identify this as the most successful approach for secondary fracture prevention [11, 15–18]. In this literature survey, we investigate what has been published on components of the bone- and fall-related risk factor phenotype in patient attending the FLS.

## Methods

A literature search was conducted in PubMed/Medline, EMBASE and CINAHL to identify relevant publications up to and including October 2016 using the following search terms: Fracture Liaison Service, fracture prevention service, fracture prevention clinic, fracture prevention program, osteoporosis clinic, and secondary fracture prevention. The search was limited to human studies in adults (18–64 years) and aged (≥65 years) written in English. We specifically selected articles which reported components of the phenotype of patients at the FLS. Finally, additional relevant publications known to us were added.

## Results

### Search Results

After removing duplicates, our search resulted in 373 potentially relevant publications. Based on title and abstract screening, 270 publications were excluded. Based on full-text eligibility assessment, 80 publications were excluded, resulting in 23 being selected. The reasons for exclusion were no FLS population (*n* = 40), and no components of the phenotype reported (*n* = 40). In addition, manual searches through the reference lists were performed, resulting in 10 additional publications. In total, 33 publications were included in this literature review (Table 1).

**Table 1:** Patient selection procedure and number of identified patients, selected patients, attenders, and included attenders

Author	Country	Year	Patient identification and selection for evaluation at the FLS					Selected patients, *n*	Attenders, *n* (%)	Selection for publication^a^	Subjects, *n* (%)^b^	Subjects, %^c^
			IP/OP	F/M	Age	Fracture	Additional criteria					
Patient selection for FLS evaluation conform recommendations, all FLS attenders selected for publication (*n* = 12)												
McLellan [13]	GBR	2003	IP+OP	F+M	50+	All	1	4671				
Blonk [19]	NLD	2007	IP+OP	F+M	50+	All	1, 2, 5	1,220	1058 (87)		1,058 (100)	87
Eekman [20]	NLD	2014	IP+OP	F+M	50+	All	1, 9	2,207	1116 (51)		1,116 (100)	51
Fraser [21]	AUS	2016	IP+OP	F+M	50+	All	1	841	166 (20)		166 (100)	20
Malgo [22]	NLD	2016	IP+OP	F+M	50+	All	2, 3, 5, 6, 7	856	709 (83)		709 (100)	83
Naranjo [23]	ESP	2014	IP+OP	F+M	50+	All	1, 2, 6	532	330 (62)		330 (100)	62
Naranjo [24]	ESP	2015	IP+OP	F+M	50+	All	1, 2, 6	1324	759 (57)		759 (100)	57
Ojeda [25]	ESP	2010	IP+OP	F+M	50+	All	1, 2, 6	683	380 (56)		380 (100)	56
Woltman [26]	NLD	2010	IP+OP	F+M	50+	All	1,2		523		523 (100)	
Ong [27]	GBR	2014	IP+OP	F+M	50+	All	1		4288		4,288 (100)	
Van den Berg [28]	NLD	2014	IP+OP	F+M	50+	All	8		1898		1,898 (100)	
Huntjens [29]	NLD	2011	IP+OP	F+M	50+	All	1, 2, 5		7199		7,199 (100)	
Patient selection for FLS evaluation conform recommendations, subgroup of FLS attenders selected for publication (*n* = 12)												
Bours [30]	NLD	2011	IP+OP	F+M	50+	All	1, 2, 3	893	656 (73)	a	626 (95)	70
De Klerk [31]	NLD	2012	IP+OP	F+M	50+	All	1, 2, 5		194	a	176 (91)	
De Klerk [32]	NLD	2013	IP+OP	F+M	50+	All			541	a	499 (92)	
Hegeman [33]	NLD	2004	IP+OP	F+M	50+	All	1, 5	156	116 (74)	a	100 (86)	64
Wyers [34]	NLD	2014	IP+OP	F+M	50+	All	1, 2, 3	3057	1694 (55)	a	1,359 (80)	44
Van Helden [35]	NLD	2008	IP+OP	F+M	50+	All	2, 8, 9	797	708 (89)	a	568 (80)	71
Van Helden [36]	NLD	2007	IP+OP	F+M	50+	All	2, 5, 8, 9	425	288 (68)	a, b	277 (96)	65
Langridge [37]	GBR	2007	IP+OP	F+M	50+	All	1			c	2,489	
Dumitrescu [38]	NLD	2008	IP+OP	F+M	50+	All	2, 8	1013	590 (58)	a, d	100 (17)	10
Gallacher [39]	GBR	2007	IP+OP	F+M	50+	All	1			a, e	337	
Howat [40]	GBR	2007	IP+OP	F+M	50+	All	1			e	577	
Gallacher [41]	GBR	2005	IP+OP	F+M	50+	All	1			d, e	50	
Patient selection not conform recommendations (*n* = 9)												
Huntjens [42]	NLD	2013	IP+OP	F+M	50+	NVF	2, 9		834		834 (100)	
Ahmed [43]	IRL	2012	IP+OP	F+M	45+	All	1	158	124 (78)		124 (100)	78
Abbad [44]	FRA	2016	IP+OP	F+M	75+	All	1	176	110 (64)		110 (100)	64
Premaor [45]	GBR	2010	IP+OP	F	PM	All	1		1641		1,641 (100)	
Premaor [46]	GBR	2010	IP+OP	F	PM	All	1		1641	f	1,005 (61)	
Dehamchia [47]	FRA	2014	IP	F+M	No limit	All	1, 5, 8, 9	872	338 (39)	g	335 (99)	38
Nassar [48]	FRA	2014	IP	F+M	50+	NVF	1, 2, 5		528	a	362 (69)	
Ganda [49]	AUS	2015	NR	F+M	45+	All	1, 2, 4, 10	828	560 (68)	b	234 (42)	28
Beringer [50]	GBR	2006	NR	F+M	No limit	All	1			a, h	86	

1: HET, 2: Pathological fracture, 3: Periprosthetic fracture, 4: Metabolic bone disorder, 5: Cognitive impairment, 6: Poor medical status/severe functional disability, 7: Patients who had osteoporosis screening in another hospital, 8: Already on osteoporosis treatment, 9: Patients residing outside the hospital’s postal area, 10: Nursing home or hostel residence

^a^ a: All assessments completed, b: Follow-up data, c: Patients aged ≥65 years, d: Patients with osteoporosis, e: Patients with NVF, f; Patients aged <75 years, g: Pregnant women (*n* = 1), patients with primary hyperparathyroidism (*n* = 2), h Non-hip fracture patients

^b^ Presented as % of FLS attenders

^c^ Presented as % of patients selected for evaluation at the FLS

### Patient Selection Procedure

The patient selection procedure can comprise up to three steps: (1) the identification and selection of patients with a recent clinical fracture for evaluation at the FLS, (2) the patients’ response to the FLS invitation (i.e. the proportion of patients willing and able to attend the FLS), and optionally (3) the selection of a subgroup of FLS attenders to be included in the publication.

### Identification and Selection of Patients for Evaluation at the FLS

**Fig. 1:**
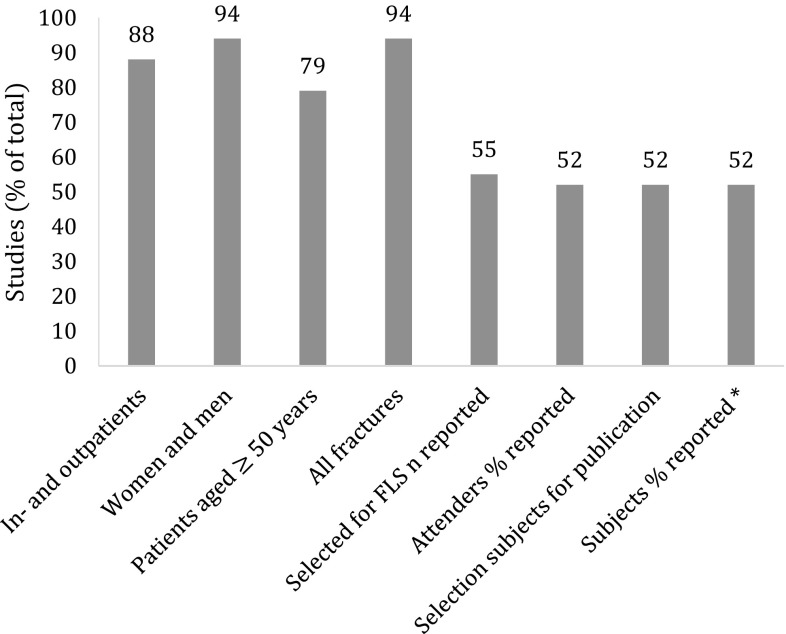
Percentage of studies reporting aspects of patient selection. * subjects as percentage of patients selected for evaluation at the FLS

Patient identification and selection differed markedly across studies (Table 1 and Fig. 1). Twenty-nine studies identified and selected in- and outpatients [13, 19–46], two studies selected only inpatients [47, 48], and two did not report this aspect of patient identification and selection [49, 50]. With respect to age, 26 studies identified and selected patients age 50 years or older [13, 19–42, 48]. Five studies used other age criteria, namely patients aged 45 years and older [43, 49], patients aged 75 years and older [44], or those who were postmenopausal [45, 46]. In two studies, no age criterion was used [47, 50]. Thirty-one studies identified and selected both men and women [13, 19–44, 47–50], whereas two studies selected only postmenopausal women [45, 46]. Patients with any fracture were identified and selected in 31 studies [13, 19–41, 43–47, 49, 50], whereas only patients with a non-vertebral fracture were selected in two studies [42, 48].

Various additional exclustion criteria were used, such as high energy trauma fractures, pathological fractures and cognitive impairment. The total number of patients identified and selected for evaluation at the FLS was reported in 18 (55%) of 33 studies (Fig. 1) [13, 19–25, 30, 33–36, 38, 43, 44, 47, 49], and ranged from 156 to 3057 patients (Table 1).

### Attendance

**Fig. 2:**
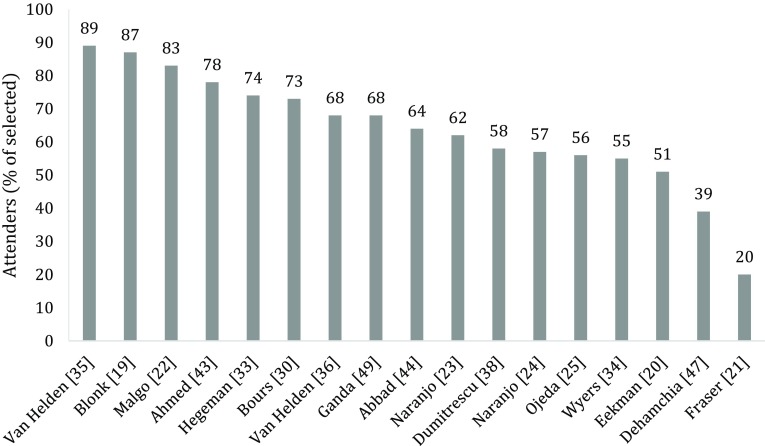
Patients attending the FLS as percentage of patients selected for evaluation at the FLS, reported in 17 studies

Selected patients were informed personally or through an information letter, except for the study by Fraser et al. [21], in which a letter was sent to the general practitioner informing them of the fragility fracture and invited referral to the fracture prevention clinic. In 17 (52%) of the 33 studies (Fig. 1), 20–89% of the patients selected for evaluation at the FLS actually attended the FLS (Table 1 and Fig. 2) [19–25, 30, 33–36, 38, 43, 44, 47, 49].

### FLS Attenders Included in the Publication

**Fig. 3:**
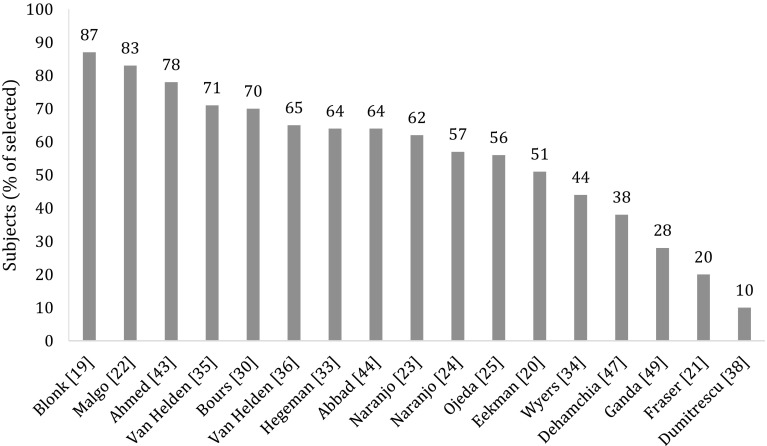
Patients selected for publication (subjects) as percentage of patients selected for evaluation at the FLS, reported in 17 studies

Of the 33 studies, 16 (48%) included all FLS attenders [13, 19–29, 42–45], whereas 17 (52%) included a subgroup of the attenders (Fig. 1): patients aged <75 years [46], patients aged 65 years or older [37], patients diagnosed with osteoporosis [38, 41], patients who completed all assessments [30–36, 38, 39, 48, 50], and those of whom follow-up data were available [36, 49]. In 12 of the 17 studies that included a subgroup, the study population was composed of 20–99% of patients attending the FLS (Table 1) [30–34, 36, 38, 46–49]. Seventeen (52%) of the 33 studies reported patients included in the study as percentage of those selected for evaluation at the FLS (Fig. 1). As a result of patients identification and selection, and study inclusion criteria, the study population was composed of 10–87% of those selected for evaluation at the FLS (Table 1 and Fig. 3).

## Components of the Phenotype

### Age and Gender

In 29 of the 31 studies in which both men and women were included, the proportion of men ranged from 13 to 30% (Table 2) [19–36, 38–44, 47–50]. As shown in Table 2, 25 of those 31 studies reported mean age, ranging from 64 to 80 years [19–27, 29, 31–38, 41, 42, 44, 47–50]. Mean age was also reported separately for men and women, ranging from 63 to 70 years in men [28, 30, 34, 35, 40, 43, 50] and from 62 to 77 years in women (Table 2) [28, 30, 34, 35, 40, 43, 45, 46, 50]. The proportion of patients aged 50–59, 60–69, 70–79, and ≥80 years were, respectively, 33–35%, 32–35%, 23–27%, and 6–9% [19, 34]. In both men and women, mean age was highest in hip fracture patients [40].

**Table 2:** Reported components of the FLS patients’ phenotype

Author	Age (mean)	Men (%)	Fracture location (%)			BMI (mean)
			Hip	Clinical VF	NV/NH	
IP+OP, F+M, 50+, all Fx						
McLellan [13]						
Blonk [19]	64	24	9	5	86	27
Eekman [20]	68	22				
Fraser [21]	70	14	8	10	82	
Malgo [22]	67	27	9	6	85	
Naranjo [23]	71	23	22	6	72	
Naranjo [24]	72	22	26			
Ojeda [25]	70	13	19	8	73	29
Woltman [26]	73	21	23	2	75	
Ong [27]	66	17				
Van den Berg [28]		20				
Huntjens [29]	67	23	6			
Bours [30]		23				
De Klerk [31]	67	21	8	13	79	28
De Klerk [32]	66	22				
Hegeman [33]	67	26	11	3	86	25
Wyers [34]	65	28	8			26
Van Helden [35]	67	28	13	3	84	
Range	64–73	13–28	6–26	2–13	72–86	25–29
IP+OP, F+M, 50+, NVF						
Gallacher [39]		23	5	Excl.	95	24
Howat [40]		21	13	Excl.	87	
Huntjens [42]	67	27		Excl.		
Range	67	21–27	5–13	Excl.	87–95	24
IP+OP, F+M, all Fx, various ages						
Langridge [37]	78		28			
Ahmed [43]		19	2	3	95	
Abbad [44]	80	21	45			
Miscellaneous						
Van Helden [36]	67	28				
Dumitrescu [38]	68	27	17	4	79	
Gallacher [41]	66	24	26	Excl.	74	
Premaor [45]		Excl.	6	<1	94	27
Premaor [46]		Excl.	10	<1	90	27
Dehamchia [47]	67	25	28			
Nassar [48]	74	13	51	Excl.	49	24
Ganda [49]	65	20				
Beringer [50]	65	30	Excl.	41	59	
Range overall	64–80	13–30	2–51	<1–41	49–95	24–29

### Fracture Location

In 23 of the 32 studies that included hip fracture patients, the percentage of patients that had a hip fracture ranged from 2 to 51% (Table 2) [19, 21–26, 29, 31, 33–35, 37–41, 43–48]. In 14 of the 28 studies that included patients with a clinical vertebral fracture, the percentage of patients with this fracture was reported, ranging from <1 to 41% (Table 2) [19, 21–23, 25, 26, 31, 33, 35, 38, 43, 45, 46, 50]. Most common were non-vertebral, non-hip (NVNH) fractures, of which the prevalence was reported in 18 of the 33 studies, ranging from 49 to 95% (Table 2) [19, 21–23, 25, 26, 31, 33, 35, 38–41, 43, 45, 46, 48, 50]. Distal radius/ulna fractures were reported as the most common NVNH fracture (27–32%) [13, 22, 39, 47], followed by humeral fractures (11–31%) [13, 22, 39, 47], ankle fractures (11–16%) [13, 22, 39, 47], and hand and foot fractures (6–16%) [13, 39]. Analyses for men and women separately showed that distal radius/ulna fractures were most common in women (21.8–38.7%), whereas hand (19.7%) [19], and ankle fractures [40] were most common in men. In three studies [29, 30, 34], fractures were classified according to Center et al. [6]. Hip fractures were present in 1–8% of patients, major fractures in 13–33%, minor fractures in 58–79%, and finger or toe fractures in 1–13%.

### Body Mass Index

Mean body mass index (BMI) was reported in nine studies, ranging from 24 to 29 kg/m^2^ (Table 2) [19, 25, 31, 33, 34, 39, 45, 46, 48], and was similar for men and women [30, 34]. According to the World Health Organisation BMI classification, 2–6% of patients were classified as underweight (<18.50 kg/m^2^), 31–33% had a normal BMI (18.50–24.99 kg/m2), 35–38% were overweight (25.00–29.99 kg/m^2^), and 26–30% were obese (≥30 kg/m^2^) [27, 39, 46].

### Bone Mineral Density

**Fig. 4:**
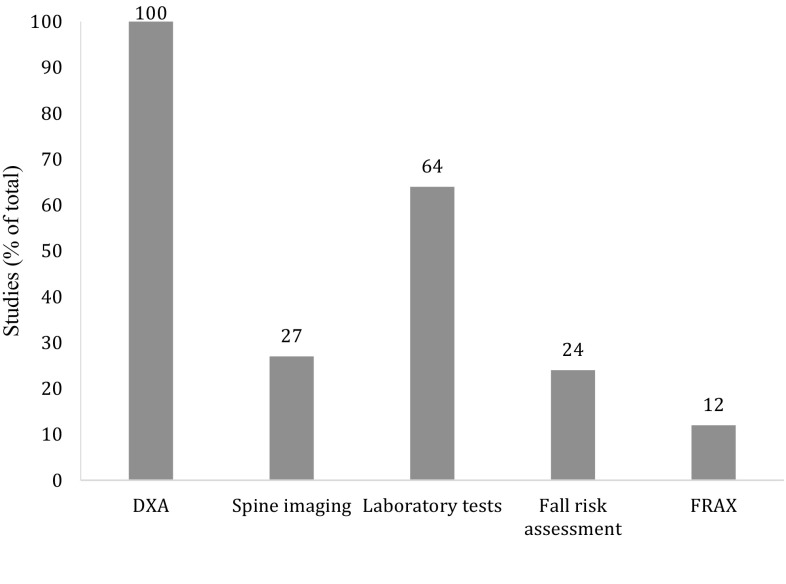
Percentage of studies reporting assessments for fracture risk evaluation

In all 33 studies, bone mineral density (BMD) measurement at the lumbar spine and hip was performed (Table 3 and Fig. 4) [13, 19–50], with additional measurements at the distal radius in one study [33]. Based on the lowest T-score, osteoporosis was diagnosed in 12–54% of patients in 22 studies [19, 21–24, 26, 28–36, 39, 43–48], osteopenia was diagnosed in 29–55% of patients in 18 studies [21–24, 29–36, 39, 43–47], and 13–39% of patients had a normal BMD in 18 studies [21–24, 29–36, 39, 43–47]. Osteoporosis was reported in 14–43% of women and in 6–28% of men [13, 28–30, 32, 34, 35]. Osteoporosis was most common in patients with a hip (36–63%) [13, 19, 29, 48], and vertebral fracture [19], and least in patients with a foot, and clavicle fracture [19]. Classified according to Center et al. [6], osteoporosis was found in 31% of patients with a minor, in 49% of patients with a major, and in 58% of patients with a hip fracture [30]. Osteopenia was found in 49% of patients with a minor, in 39% of patients with a major, and in 42% of patients with a hip fracture [30].

### Vertebral Fracture Assessment

Imaging of the spine was performed using densitometric vertebral fracture assessment (VFA) in four studies [38–40, 48], and X-ray in five (Table 3 and Fig. 4) [19, 28, 31, 33, 44]. Classified according to Genant et al. [51], vertebral fractures (VF) were present in 20–57% of patients [31, 33, 38–40, 44, 48], with VF grade 2 or 3 in 55–73% of VF patients and 17–31% of all patients [38, 39, 48]. The prevalence of VF was similar for men (19–24%) and women (20–25%) [39, 40]. VF were present in 30% of non-vertebral fracture patients aged >75 years compared with 23 and 22% of patients aged 50–64 years and 65–75 years [39]. In contrast, Howat et al. [40] reported higher prevalence rates of VF with increasing age. The prevalence of VF varied by NVF location, with highest prevalence in hip fracture patients for both men (hip fractures 32% vs. ankle fractures 8%) and women (hip fractures 31% vs. humeral fractures 5%) [39, 40, 48]. Patients with lumbar spine T-scores in the osteoporotic range were more likely to have VF (42%) than patients with T-scores in the osteopenic or normal range (20 and 16% respectively (*p* < 0.05)) [39]. Similar findings were reported for VF grade 2 or 3 (34 vs. 13 vs. 9% of patients with osteoporosis, osteopenia, and a normal BMD, respectively (*p* < 0.0001)) [39].

### Trabecular Bone Score

Only Nassar et al. [48] reported the trabecular bone score (TBS) in non-vertebral fracture patients at the FLS. Mean TBS was 1.201 ± 0.113 and mean TBS was lower in patients with VFs than in those without VFs in VFA (1.156 ± 0.108 vs. 1.227 ± 0.107, *p* < 0.0001).

### Laboratory Tests

Performance of laboratory test to investigate contributors to secondary osteoporosis and metabolic bone disorders (SECOB) was reported in 21 studies (Fig. 4) [13, 19–25, 28–30, 32–34, 37–39, 41, 43, 47, 50]. Two studies reported contributors to SECOB including vitamin D deficiency (<50 nmol/L), ranging from 50 to 70% [30, 38], and three studies reported contributors to SECOB excluding vitamin D deficiency, ranging from 3 to 28% (Table 3) [22, 30, 32]. The prevalence rates of contributors to SECOB were similar for men and women (28 vs. 26%) [30], were higher in patients with osteoporosis (33–35%) compared to 27–29% and 10–18% of those with osteopenia and a normal BMD, respectively [22, 30] and were also higher in patients with more severe fractures according to Center (23).

**Table 3:** Performance of assessments (DXA, VFA, laboratory tests, and fall risk assessment), and when reported, the results

Author	DXA	Normal BMD (%)	Osteopenia (%)	Osteoporosis (%)	VFA	Any VF (%)	Grade 2/3 VF (%)	Lab	SECOB (%)	Vit. D def. (%)	Fall risk assessment	Fall risk (%)
IP+OP, F+M, 50+, all Fx												
Blonk [19]	+			37	+			+			–	
Van den Berg [28]	+			12	+			+			–	
Hegeman [33]	+	23	44	33	+	22		+		69	–	
De Klerk [31]	+	35	38	27	+	42		–			–	
Huntjens [29]	+	21	47	32	–			+			+	
McLellan [13]	+				–			+			–	
Eekman [20]	+				–			+			–	
Fraser [21]	+	19	45	36	–			+			–	
Malgo [22]	+	17	55	28	–			+	28	43	–	
Naranjo [23]	+	20	38	43	–			+			–	
Naranjo [24]	+	13	44	42	–			+			–	
Ojeda [25]	+				–			+			–	
Bours [30]	+	15	46	30	–			+	27^a^, 70^b^	64	–	
De Klerk [32]	+	30	49	21	–			+	3/11^c^		–	
Wyers [34]	+	23	48	30	–			+			–	
Van Helden [35]	+	21	44	35	–			–			+	75
Woltman [26]	+			46	–			–			–	
Ong [27]	+				–			–			–	
Range		13–35	38–55	12–46		22–42			3–70	43–69		75–80
IP+OP, F+M, 50+, NVF												
Gallacher [39]	+	35	37	28	+	25	17	+			–	
Howat [40]	+				+	20		–			+	
Huntjens [42]	+				–			–			+	60
Range		35	37	28		20–25	17					60
IP+OP, F+M, all Fx, various ages												
Abbad [44]	+	17	29	54	+	40		–			+	
Langridge [37]	+				–			+			+	
Ahmed [43]	+	33	38	29	–			+		64	–	
Miscellaneous												
Dumitrescu [38]	+	Excl.	Excl.		+	57	31	+	50^b^	62	+	79
Nassar [48]	+			52	+	37	21	–			–	
Gallacher [41]	+	Excl.	Excl.		–			+		72	–	
Dehamchia [47]	+	19	45	36	–			+			–	
Beringer [50]	+				–			+		56	–	
Van Helden [36]	+	24	47	29	–			–			+	84
Premaor [45]	+	39	41	19	–			–			–	
Premaor [46]	+	39	41	19	–			–			–	
Ganda [49]	+				–			–			–	
Range overall		13–39	29–55	12–54		20–57	17–31		3–70	43–72		60–84

Four studies [21, 38, 41, 50] reported mean vitamin D, ranging from 44 to 68 nmol/L and seven studies [22, 30, 33, 38, 41, 43, 50] reported vitamin D <50 nmol/L, ranging from 42 to 72% (Table 3). Mean vitamin D was lower in hip than in non-hip fracture patients (35 vs. 48 respectively, *p* = .019) [41]. The prevalence of vitamin D <50 nmol/L was similar for men and women (62 vs. 53% respectively, *p* = .478) [50], for patients aged <75 years and those aged ≥75 years (53 vs. 61% respectively, *p* = .522) [50], and for patients with osteoporosis, osteopenia and a normal BMD (42 vs. 43 vs. 42% respectively) [22].

### Daily Calcium Intake

Only three studies reported mean daily calcium intake [19, 33, 38], ranging from 759 to 912 mg/day, and two studies reported daily calcium intake <1200 mg/day, ranging from 86 to 91% of patients [30, 38]. Daily calcium intake <1200 mg/day was similar for men and women, age decades, fracture location according to Center et al. [6], and patients with a normal BMD, osteopenia, and osteoporosis [30].

### Fracture Risk Assessment Tools

FRAX score for major fractures was 8–13% in four studies, and for hip fractures 3–7% in four studies [23–25, 28]. In 46–49% of patients, FRAX score for hip fractures was >3% [23, 24].

### Fall-risk Assessment

Fall-risk assessment was reported to be performed in eight studies (Fig. 4) [29, 35–38, 40, 42, 44]. Only four studies [35, 36, 38, 42] reported prevalence rates of fall-risk factors, with at least one fall-risk factor in 60–84% of patients (Table 3). All fall-risk factors were more frequently reported in women, with the exception of impaired vision, which was found in 25% of women and 31% of men [35].

## Discussion

This survey aimed to describe the bone- and fall-related components of the phenotype of patients attending the FLS based on 33 FLS related papers. The reported phenotypic characteristics varied widely among the various publications with regard to the mean age, proportion of men, and fracture location. In addition, the proportion of patients with osteoporosis, prevalent vertebral fractures, newly diagnosed contributors to secondary osteoporosis and metabolic bone disease, and proportion of patients with fall-related risk factors varied substantially across studies. Although, there is a great heterogeneity in components of the phenotype, the prevalence rates of these components were high.

The heterogeneity of reported phenotypes of FLS patients can be explained by several aspects. Firstly, the variability in the FLS patients phenotype can be explained by differences in patient selection and FLS attendance. Positioning papers on secondary fracture prevention by the ASBMR, IOF, and EULAR/EFORT [11, 15, 18], recommended that all patients aged 50 years or older with a recent fracture should have their risk for subsequent fractures evaluated at the FLS. In three out of four studies, this recommendation was implemented successfully. Nine studies selected another group of patient for evaluation at the FLS based on different selection criteria (only inpatients, only women, only patients aged 75 years or older, only NVF patients). Additionally, various combinations of selection criteria were used, such as only low-trauma or fragility fracture patients, or excluded patients with pathological fractures. Further, FLS attendance rates ranged from 20 to 89%. This indicates that achieving adequate FLS patient selection and attendance is a major challenge and often hampered by logistic obstacles. It has been shown that FLS care with a central coordinator (often a specialised nurse) is the most appropriate clinical organization model for secondary fracture prevention [11, 15, 18]. Although capturing all fracture patients is the ultimate goal, it has been suggested that an FLS may initially focus on a subgroup [15]. Once secondary fracture prevention for these patients has been well-established, the scope of the FLS should be expended to eventually include all fracture patients. In addition, other approaches, such as an orthogeriatric service, may have been established in hospitals to systematically optimise care of hip fracture patients, including components covered by a FLS [52]. This type of service of course alters the phenotype of the patients attending the FLS. In our literature survey, all but six studies focussed on all patients regardless of their fracture location. Of these six studies, one study [50] excluded hip fractures.

Second, as recommended in the positioning papers, risk evaluation should include dual-energy X-ray absorptiometry (DXA), and vertebral fracture assessment (VFA), and on indication, laboratory tests, and fall risk assessments [18]. DXA evaluation was performed in all studies, imaging of the spine in nine studies, laboratory tests in 21 studies, and fall risk evaluation in eight studies. Since these assessments often have to be justified through local business cases supported by solid health economic analysis, which are currently lacking, implementation of these assessments is not always feasible. Hence, the reported outcomes of the various bone- and fall-related components of FLS patients may be influenced not only by patients selection and attendance rates, but also by the possibility to perform additional assessments in all FLS patients [52].

Based on these results in literature, it is difficult to describe the full spectrum of bone and fall risks in patients attending the FLS. In the context of fracture prevention, knowledge of the presence and combinations of the risk factors will guide the need for evaluation and treatment. In this literature survey of FLS, we found a high variability in patient selection and fracture risk evaluation. In order to specify the bone- and fall-related phenotypes at the FLS, systematic studies on the presence and combinations of these risks are needed.
